# Evaluation of four commercial real-time RT-PCR kits for the detection of dengue viruses in clinical samples

**DOI:** 10.1186/1743-422X-11-164

**Published:** 2014-09-15

**Authors:** Fatiha Najioullah, Florent Viron, Raymond Césaire

**Affiliations:** Laboratoire de Virologie, Centre Hospitalier Universitaire de Fort-de-France, and EA 4537, Université des Antilles et de la Guyane, Martinique, France

**Keywords:** Dengue, Diagnosis, Real-time polymerase chain reaction, RT (reverse transcription)-PCR, Genotype, Quantification, Commercial assays

## Abstract

**Background:**

Dengue is the most frequent arthropod-borne viral disease worldwide. Because dengue manifestations are similar to those of many other febrile syndromes, the availability of dengue-specific laboratory tests is useful for the differential diagnosis. Timely and accurate diagnosis of dengue virus (DENV) infection is important for appropriate management of complications, pathophysiological studies, epidemiological investigations and optimization of vector-control measures. Several “in-house” reverse transcriptase-polymerase chain reaction (RT-PCR) methods have been developed to detect, type and/or quantify DENV. Standardized dengue RT-PCR kits with internal controls have been recently introduced, but need clinical evaluation. We assessed the performances of 4 commercial DENV real-time RT-PCR kits.

**Findings:**

The 4 kits were evaluated using a panel of 162 samples positive with an existing in-place hemi-nested RT-PCR used for routine DENV-infection diagnosis in patients with acute-febrile disease. The panel included 46 DENV-1, 37 DENV-2, 33 DENV-3, and 46 DENV-4. Also, 70 negative serum specimens were used to determine specificity. Geno-Sen’s Dengue 1–4 Real-Time RT-PCR kit was the only assay to provide quantification using standards, but lacked sensitivity for DENV-4 detection. The Simplexa^TM^ Dengue RT-PCR assay, with 151 (93.2% [95% confidence interval, 89.3–97.1]) positive samples, had significantly higher sensitivity than the other 3 kits; in a complementary evaluation of 111 consecutive patients’ samples, its performance and genotyping agreed with the hemi-nested gold-standard assay.

**Conclusions:**

The Simplexa^TM^ Dengue RT-PCR’s good performance to detect and genotype DENV1–4 requires further evaluation in multicenter and prospective studies, particularly in settings of clinical diagnosis during dengue outbreaks.

## Findings

Dengue is caused by 4 related viruses, (DENV)-1, -2, -3 and -4, and is the most common and widespread arthropod-borne viral disease in the world. This self-limited infection can be asymptomatic or cause a clinical spectrum ranging from mild fever (DF) to severe, life-threatening illness, dengue hemorrhagic fever (DHF) and dengue shock syndrome (DSS). Other severe manifestations, including hepatitis, myocarditis or encephalitis, may occur independently of plasma leakage [1]. Because dengue symptoms are similar to those of many other undifferentiated febrile syndromes, new commercial tests that can be used for case management have to be clinically evaluated. Laboratory-based surveillance is also of importance for early warning of dengue outbreaks and optimization of vector-control measures [2].

Current diagnostic methods have limitations. Enzyme-linked immunosorbent assays to detect immunoglobulin M are used by most laboratories, but that response is absent early during the disease course and may remain undetectable in secondary dengue. DENV-NS1 antigen can be detected during the first few days of fever. However, recent evaluations of commercial NS1 assays have had limited sensitivity [3, 4]. Virus isolation from acute-phase sera is useless for patient management, because several days are needed to obtain final results. Molecular methods yield same- or next-day detection of DENV in acute-phase serum or plasma. The 2-step hemi-nested reverse transcriptase-polymerase chain reaction (RT-PCR) protocol, originally reported by Lanciotti et al. [5] and later modified to a single-step multiplex RT-PCR [6], has proved to be highly sensitive [7], and is used worldwide. Conventional RT-PCR is being progressively replaced by real-time RT-PCR, which limits the risks of carryover contamination and is suitable for DENV-genome detection. Another advantage of real-time RT-PCR is that it yields reliable quantification, making it a useful tool for pathophysiological studies. Almost all of the reported RT-PCR methods are “in-house” procedures [8–29]. International external quality studies have highlighted sensitivity and specificity heterogeneities, even inter-laboratory using the same method [30].

Levi et al. [31] evaluated a commercial real-time RT-PCR kit (RealArt; artus/Qiagen, Germany) and found high agreement with an in-house multiplex RT-PCR, but the test was limited to DENV-3, and occasionally DENV-2, samples. More recently, the US Food and Drug Administration approved a Centers for Disease Control DENV-1–4 real-time RT-PCR assay [32], but it has been shown to be less sensitive than a laboratory-developed assay, particularly for DENV-1 [33]. Herein, we evaluated the performances of 4 recently developed commercial DENV real-time RT-PCR kits.

The study was conducted in the Virology Laboratory, where biological samples from patients with acute-febrile syndromes are processed as follows: a 5 ml venous blood sample, collected on the day of admission, is centrifuged, and plasma or serum supernatant is divided into 2 aliquots, one stored at -4°C for daily RT-PCR and the other frozen at -80°C until use. We analyzed 162 laboratory-confirmed stored samples collected during the outbreaks that occurred the last decade: 46 DENV-1, 37 DENV-2, 33 DENV-3, and 46 DENV-4. A panel of 70 negative specimens was used to determine specificity. In addition, we prospectively tested 111 samples from patients consecutively seen at the emergency unit for acute-febrile syndromes at the peak of the 2010–2011 epidemic.

Because the study was non-interventional, e.g. with no additional samplings or specific procedures for subjects, no consent form from the patient was needed, as stated by the French Public Health code. The consecutive samplings were part of routine diagnosis. The dengue sample collection has been declared under the number DC-2009-979 to the French ministry of health and the “Comité de protection des personnes Bordeaux sud-ouest/Outre-mer”, according to the Bioethics laws (décret n°2007-1220 du 10 août 2007 relatif aux activités de prélèvement et conservation à des fins scientifiques de collections d’échantillons biologiques). The dengue collection is stored at the “Centre de Ressources Biologiques de la Martinique” (CeRBiM). The CeRBiM database has been agreed by the CNIL (“Commission nationale de l’informatique et des libertés”).

RNA was extracted from 200 μL of stored aliquots using NuCliSens EasyMag (bioMérieux, Craponne, France). All samples were screened by hemi-nested RT-PCR with the dengue virus consensus primers D1 and D2 and the serotype-specific primers TS1, TS2, TS3, and TS4 [5]. Lanciotti’s protocol was adapted using the SuperScript One-Step RT-PCR System with Platinum *Taq* DNA Polymerase (Invitrogen, Life Technologies, France) [34]. Briefly, the first-round reaction was performed with primers D1 and D2, 1 μl of SuperScript II RT/Platinum Taq mix (Invitrogen), and 5 μl of the RNA template. The second amplification was used forward primer D1 and downstream primers TS1, TS2, TS3, and TS4, with the TaqPCR Master Mix kit (Qiagen). Both the RT-PCR and nested-PCR products were analyzed by gel electrophoresis on a 2% agarose gel with ethidium bromide for ultraviolet visualization. The expected size of the primary PCR amplicons was 511 bp. Nested-PCR bands of 482, 119, 290, and 389 bp were considered specific for DENV-1, DENV-2, DENV-3, and DENV-4, respectively. The sensitivity of the assay has been previously evaluated to 1 PFU/ml with dengue virus titrated controls, and 1–10 molecules/reaction with dengue virus RNA standards obtained by cloning the capsid-prM region into pCR4-TOPO vector (data not shown). Finally, the procedure was validated through participation in a multicenter quality-control study conducted by the French National Reference Center for Arboviruses (Institut de Recherche Biologique des Armées, France).

Four commercial kits were evaluated versus Lanciotti’s gold-standard protocol. All the commercial kits were fully furnished, and were used according to the manufacturer’s recommendations. The characteristics of the 4 kits tested are summarized in Table 1.

**Table 1:** **Characteristics of 4 commercial DENV real-time RT-PCR kits**

Characteristic	Geno-sen’s	Liferiver	Realstar	Simplexa
Target region	Not specified	Not specified	Not specified	DENV-1 NS5
				DENV-2 NS3
				DENV-3 NS5
				DENV-4 capsid
Principle	TaqMan	TaqMan	TaqMan	TaqMan
Dye	FAM	FAM	FAM	FAM/CFR610
Internal control (dye)	Yes (JOE)	Yes (JOE)	Yes (JOE)	Yes (Q670)
PCR reactions, *n*	1	1	1	2
Genotyping	No	No	No	Yes
Quantitative	Yes	Yes	No	No
External standards	10^4^–10^8^ copies/mL	10^7^ copies/mL	Yes (not quantified)	Yes (not quantified)

### Simplexa^TM^dengue RT-PCR assay *(Focus Diagnostics, Cypress, CA)*

The assay is based on bi-functional fluorescent-probe primers and reverse primers amplifying *DENV-1 NS5*, *DENV-2 NS3*, *DENV-3 NS5*, and *DENV-4 capsid* genes. The Simplexa dengue 1-and-4–primer mix contains a DENV-1–fluorescent 6-carboxyfluorescein (FAM) probe and a DENV-4–CFR610 probe. The Simplexa dengue 2-and-3–primer mix contains a DENV-3–FAM probe and a DENV-2–CFR610 probe. Each primer mix includes a Q670-labeled probe to detect the RNA internal control (IC) used to monitor the extraction process and RT-PCR inhibition. A positive control is provided in the kit.

### RealStar dengue RT-PCR kit 1.0 *(Altona Diagnostics, Hamburg, Germany)*

This assay is based on the amplification of DENV-specific target sequences and target-specific probes labeled with FAM reporter and quencher dyes. The probe specific to the IC target is labeled with the fluorophore JOE. The IC is added to the specimen/lysis buffer mixture. Primers and probes, based on 2010 sequence alignments of all 4 DENV, detect but do not genotype them.

### Dengue virus general type real-time RT-PCR kit Liferiver^TM^*(Shanghai ZJ Bio-Tech Co, China)*

This kit contains a super mix for the specific amplification of DENV-1–4 RNA. DENV amplicons are detected using a probe carrying a FAM fluorophore and BHQ1 quencher. The kit contains a system to identify possible RT-PCR inhibition by measuring the VIC/JOE fluorescence of the IC. The supplied external positive control, defined as 1 × 10^7^ copies/mL, enables determination of the gene load.

### Geno-Sen’s dengue 1–4 real-time RT-PCR kit *(Genome Diagnostics Pvt, New Delhi, India)*

The specific master mix contains reagents and enzymes for the specific amplification of DENV-1–4 and the direct detection of the specific amplicon using the TaqMan principle and FAM channel. An IC is provided to detect inhibitors in the extract. Five external positive standards, range 10^4^–10^8^ copies/mL, are supplied for determination of the gene load. The quantitation standards provided in the kit are treated in the same way as extracted samples. This method was initially designed for use in the Rotor Gene Thermocycler and we adapted it to an ABI 7500 apparatus.

In accordance with conventional clinical pratice, amplification and detection were performed in duplicate, using an ABI Prism® 7500 (Applied Biosystems, France) for LifeRiver, Real Star and GenoSen’s kits. For Simplexa, we used the 3 M integrated cycler provided by Focus. Negative and extraction controls were added at each step to avoid contamination and eliminate inhibition.

The Liferiver kit had poor sensitivity on the initial panel of 40 positive samples, detecting only 28 of them, and was not further evaluated. The Geno-Sen’s, RealStar and Simplexa kits were tested with a panel of 162 sera, and were positive for 138 (85.2%), 135 (83.3%), and 151 (93.2%), respectively (Table 2). Simplexa sensitivity was significantly higher than those of Geno-Sen’s (chi-square, *P* = 0.02) and RealStar kits (*P* = 0.006). Simplexa sensitivities were homogeneous (range 90.9%–95.6%) from one serotype to another. For all positive samples, serotype identification was concordant with Lanciotti’s hemi-nested round. RealStar achieved adequate sensitivity for DENV-3 and DENV-2 samples, but failed to detect one-fifth of DENV-1 and DENV-4 samples.

**Table 2:** **Sensitivities of commercial DENV real-time RT-PCR assays against a panel of clinical samples positive with hemi-nested RT-PCR** [5, 34]

Serotype	***N***	Geno-sen’s (*n* (% [95% CI])	Realstar (*n* (% [95% CI])	Simplexa (*n* (% [95% CI])
DENV-1	46	42 (91.3 [83.2–99.4])	36 (78.3 [66.3–90.2])	44 (95.7 [89.7–100])
DENV-2	37	33 (89.2 [79.2–99.2])	32 (86.5 [75.5–97.5])	34 (91.9 [83.1–100])
DENV-3	33	30 (90.9 [81.1–100])	30 (90.9 [81.1–100])	30 (90.9 [81.1–100])
DENV-4	46	33 (71.7 [58.7–84.8])	37 (80.4 [68.9–91.9])	43 (93.5 [86.3–100])
Total	162	138 (85.2 [79.7–90.7])	135 (83.3 [77.6–89.1])	151 (93.2 [89.3–97.1])

Geno-Sen’s was the only kit to provide quantification using standards, with median virus loads of 5 × 10^7^, 1 × 10^7^, 2 × 10^7^ and 1 × 10^5^ copies/mL, for DENV-1, -2, -3 and -4, respectively. We cannot exclude that the sampling was biased towards higher loads, since it concerned symptomatic patients. Viral loads were markedly lower for DENV-4 samples. Notably, low DENV-4 loads in infections were previously reported [17, 24, 35]. Using an in-house quantification method during a DENV-1–DENV-4 co-epidemic, we confirmed finding lower DENV-4 than DENV-1 loads (data not shown). However, we cannot exclude faulty DENV-4 amplification with Geno-Sen’s kit, and its 71% detection rate is insufficient for clinical diagnoses.

The analytical sensitivity of the Simplexa kit versus Lanciotti’s procedure was also evaluated using dilutions of DENV-1–4 strains. For all dilutions tested, Simplexa assay sensitivity was similar to that of the hemi-nested RT-PCR, with differences <0.5 log_10_. The Simplexa kit was then tested prospectively on 111 successive patients’ samples. All samples positive with the hemi-nested RT-PCR (3 DENV-1, 16 DENV-2, 6 DENV-4) were also positive with the Simplexa kit, with cycle threshold below 31 cycles. One negative sample with a cycle threshold of 38 was negative with both methods after re-extraction.

Specificity was assessed using 70 samples negative with Lanciotti’s RT-PCR. All were negative with Geno-Sen’s and Simplexa kits, but 1 was weekly positive with RealStar. Since no other flavivirus is known to circulate at significant level in inhabitants of our Caribbean island [36], we did not test for cross-reactivity with clinical specimens of other member of the genus. West Nile virus lineage I and II controls (AccuType, SeraCare, Life Sciences) were checked negative with the Dengue Simplexa RT-PCR kit. The recent outbreak of chikungunya did allow us to verify the absence of cross-reactivity of the Dengue Simplexa assay with this other arbovirus (data not shown). This is of particular interest for countries threatened by its recent emergence in the Americas. Another limitation of our evaluation is the use of Caribbean DENV, which are only representative of the Americas strains and not of DENV circulating in other part of the world.

In summary, RT-PCR–based methods are widely applied to obtain clinical diagnoses of infectious diseases, and several in-house protocols have been evaluated for DENV-RNA detection. Different reagent suppliers, reaction conditions and technical skills may influence protocol reproducibility from one laboratory to another. External quality-assurance studies showed that differences in the operational procedures led to discrepant results between reference laboratories using the same in-house methods [30]. Commercial kits developed under good manufacturing practices may offer improved reliability. They correspond better to quality-insurance rules for clinical diagnoses and include IC. Manufactured DENV RT-PCR kits may provide broader access, and be less time-consuming and more cost-effective for clinical diagnoses. Our preliminary results showed good agreement between the Simplexa^TM^ Dengue RT-PCR assay and Lanciotti’s reference hemi-nested RT-PCR, currently used in our laboratory. That kit achieved >90% detection of each serotype, with clinical samples obtained between days 1 and 7 after fever onset, and concordance for genotyping. We think that the combination of automated extraction and the Simplexa^TM^ RT-PCR kit should be evaluated in multicenter and prospective studies in the context of clinical management during dengue outbreaks.

Ethical Approval was not needed for this study, in line with French regulations.

## Abbreviations

DENV: Dengue virus
RT-PCR: Reverse transcription-polymerase chain reaction
IC: Internal control.
